# The Province of Bacteria

**Published:** 1898-05

**Authors:** B. Fincke

**Affiliations:** Brooklyn


### THE PROVINCE OF BACTERIA.

B. Fincke, M. D., Brooklyn.

The question proposed by the New York Homœopathic Union for a free discussion, "What is the Province of Bacteria in Medicine?" can of course only be expected from the standpoint of a homœopathician. Hence, at first it is to be pointed out that there is a difference of the conception of disease in the allopathic and homœopathic sense.*

According to the first, it is the expression of a disturbance of the health of the organism with a distinct name, which is deduced from pathological investigations and considered as an entity for which the medicine is applied *ab usu in morbis*, according to rules laid down in the allopathic text-books.

Second. In the homœopathic sense disease is a disturbance of the life-force of the organism, consisting in the totality of the symptoms which denotes changes from health and serves as indication for the remedies to be applied according to the homœopathic law similia similibus, and in such doses as to be proportionate to the life-force of the individual.

---

* Read before the New York Homœopathic Union.

This, then, is the prime difference in the conception of disease by the two antagonistic professions. The allopathic conception is arrived at by deduction from pathological reasoning, the homœopathic by induction from the actual state of the organism in its changes from health. The former directs its efforts upon the disease of the organism as an individual irrespective of the sick person, whilst the other by the observation of the symptoms in their complex directs its efforts upon the sick person itself irrespective of the rules laid down in the allopathic text-books for combating the diagnosed individual disease.

If that is so, then it seems that there is in Homœopathy no place for the province of bacteria introduced into medicine by the allopathic school of medicine. Bacteriology is by no means placed on such a sure footing as to warrant the dictatorial ordinances sent out for the physicians to obey in its behalf. It is still in its development, and new discoveries and corrections of previous observations are constantly made which belong to the legitimate domain of scientific research, but to foist it upon the science of healing as the *sine qua non* of treatment cannot for a moment be admitted.

Bacteria, at first considered as characteristic in certain forms of disease, and declared to be the invariable causes of the various diseases, though none have as yet been found in several forms of disease, have also been found to exist under normal conditions of the organism, and hence have no right to take the high rank as the original causes of disease, and to constitute the safe guides in the selection of our remedies. The fact is that this bacteriological investigation has grown to such an enormous extent as to bring all medical treatment under its domain by the adoption of the much despised discovery of Dr. Lux, that products of disease have been found active and useful for similar diseased states of the organism, though only under the condition of their being made homœopathic by potentiation. In the course of time, strange to say, the idea underlying this discovery has sprouted far and wide under the auspices of the modern allopathic school. The inventor of the anti-toxine serum himself has frankly acknowledged as much by assigning to Hahnemann the honor of having laid the very foundation to it by his discovery of the law of healing, and he was not ashamed at the same time to hail it as a triumph of his profession that it succeeded in silencing the Hahnemannian discovery for a century, in order to save it from exploitation by the homœopathic profession, till the great man came who invented his serums for therapeutics and puts such an exploitation in practice for his own profit. We have seen enough of the effects of these serums of his to let them alone in our treatment of the sick, for we know that the true homœopathic treatment is the most efficacious, speedy, and humane in existence. It is surprising to see how the followers of Hahnemann are carried away by the numerous allopathic artifices, which last for a while and then make room for new discoveries and inventions which are owing to improved scientific facilities without contributing to the science of healing, for this is not synonymous with the science of treating diseases. The province of bacteria, therefore, lies altogether in the allopathic pathological and physico-chemical school. Consequently we have no objections to its efforts to explore this wonderful but somewhat dirty realm of nature, as they enrich the department of natural history in the pursuance of the discovery of microscopical beings begun in the earlier part of our century with the infusoria. The greater perfection of the microscope has revealed the existence of still more minute beings in the organic bodies, standing in the relation of parasites to their constituents. Whether they are of vegetable or animal nature, whether they are organized beings endowed with function of secretion, or merely propagate and decay, is not our duty to find out in our province of medicine, viz., the healing of the sick, which by Hahnemann was discovered to belong to the dynamic domain, and requires dynamic remedies. We do not, however, object when the physicians of the old school carry on such scientific investigations which have their uses in the increase of knowledge, but we decline that every such discovery should be used immediately to overturn principles which have been found unchangeable by practice, such as the Hahnemannian doctrine of Homœopathy. We see daily that the allopathic physicians do not hesitate to administer new chemical preparations, even without knowing their origin and composition, and the serums derived from poisoning animals or cultures of bacteria to the sick before knowing anything about them but a few isolated recommendations of the chemists or bacteriologists in and out of practice. A number of poor sick creatures requiring help are thus sacrificed on the alleged altar of science before the value or worthlessness of the remedies, which generally are not remedies, but poisons in more or less crude form, is experienced. Nay, it should still more be objected to the ordinances of the health authorities requiring the acceptance of such demands upon the intelligence of educated physicians, unauthorized by true science. Why should a homœopathician stoop to use such crudenesses as are now in vogue from vaccination down to baterial inoculation because they seem to spring remotely from the fountain of the old misnamed doctrine of isopathy? or because Hahnemann had an idea that cholera was conveyed by the agency of miscroscopial animalcules in the atmosphere? Did he recommend for the cure of it any isopathic remedies such as are now applied as serums and bacterial cultures? No; he recommended Camphor, Cuprum, Veratrum, the latter two in small doses, such as the bacteriologists love to ridicule. Hahnemann sounded the death-knell to the ever-repeated efforts of the old school to oppose to disease medicines as strong as the patient can bear by trying them upon the sick. He laid down the law that every medicine should be made homœopathic to the life force by potentiation, and thus be proved upon the healthy. From this source the knowledge of the remedial power of drugs is obtained, without which no remedy is to be used upon the sick. And no remedy is introduced into the great collection of materia medica pura which has not gone through the ordeal of this investigation of proving upon the healthy.

I trust after the foregoing explanation it must be admitted that the province of bacteria in disease has no place in homœopathics, and permitted in conclusion to say that it seems strange that some homœopathic physicians who adore Hahnemann and Dunham should follow unwittingly the uncertain light of this *ignis-fatuus,* viz., bacteriology in healing the sick.

*Ceterum censeo macrodosiam esse delendam.*

